# Unravelling Strain-Specific Modifications of Toxoplasma gondii tRNA and sncRNA Using LC-MS/MS

**DOI:** 10.1128/spectrum.03564-22

**Published:** 2023-04-10

**Authors:** Wei Wang, Yi Yang, Huanping Guo, Ming-Han Li, Xiao-Qing Chen, Xin-Yu Wei, Yu Chen, Hany M. Elsheikha, Xiao-Xuan Zhang

**Affiliations:** a College of Veterinary Medicine, Qingdao Agricultural University, Qingdao, People’s Republic of China; b College of Animal Science and Veterinary Medicine, Heilongjiang Bayi Agriculture University, Daqing, People’s Republic of China; c College of Animal Sciences, Zhejiang Provincial Key Laboratory of Preventive Veterinary Medicine, Institute of Preventive Veterinary Medicine, Zhejiang University, Hangzhou, People’s Republic of China; d Medical Center of Hematology, The Second Affiliated Hospital of Army Medical University, Chongqing, People’s Republic of China; e College of Veterinary Medicine, Jilin Agricultural University, Changchun, People’s Republic of China; f Jiangxi Provincial Key Laboratory for Animal Health, College of Animal Science and Technology, Jiangxi Agricultural University, Nanchang, People’s Republic of China; g Faculty of Medicine and Health Sciences, School of Veterinary Medicine and Science, University of Nottingham, Sutton Bonington Campus, Loughborough, United Kingdom; National Institutes of Health

**Keywords:** *Toxoplasma gondii*, epigenetics, RNA modification, virulence, tRNA, sncRNA

## Abstract

Many RNA modifications have been detected in rRNA, tRNA and small noncoding RNA (sncRNA) as well as in low-abundance RNA species such mRNA. Although RNA modifications play roles in many cellular and biological processes in various domains of life, knowledge about the diversity and role of RNA modifications in Toxoplasma gondii is limited. In this study, RNA modifications in three T. gondii strains (RH type I, PRU type II, and VEG type III) with distinct virulence abilities were determined by liquid chromatography-tandem mass spectrometry. We compared the levels of modifications of four nucleotides in tRNA and sncRNA, characterized RNA modification patterns of different T. gondii strains, and determined the diversity of RNA modifications. We detected and quantified 22 modified nucleosides in both tRNA and sncRNA. Significant differences in the diversity of the modified nucleosides were found between the three T. gondii strains. RNA modifications were correlated with the expression of many T. gondii virulence proteins. Some of the identified modifications (e.g., 2’-O-methylinosine, pseudouridine) play a role in mediating the host-parasite interaction. These results provide novel insight into the global modifications in tRNA and sncRNA, and the diversity of RNA modifications between T. gondii strains with different virulence backgrounds.

**IMPORTANCE** Although RNA modifications play roles in many cellular and developmental processes in various domains of life, knowledge about the patterns and functions of RNA modifications in T. gondii is limited. Here, a quantitative liquid chromatography-tandem mass spectrometry (LC-MS/MS) approach was used to study global RNA modifications in T. gondii strains of distinct virulence backgrounds. We quantified 22 modified nucleosides in both tRNA and sncRNA. Significant T. gondii strain-specific differences in RNA modifications were detected. More tRNA modifications correlated with T. gondii virulence proteins than sncRNA modifications. RNA modifications were significantly correlated with virulence proteins. Our data provide the first comprehensive profiling of the modifications tRNA and sncRNA in T. gondii, expanding the diversity of RNA modifications in this parasite and suggesting new regulators for modulating its virulence.

## INTRODUCTION

Toxoplasma gondii is a widespread protozoan parasite that infects most warm-blooded animals and roughly one-third of the world’s human population (1). Cats and other felines serve as the definitive host by supporting the formation and excretion of oocysts, which represent the main source of T. gondii infection in humans and animals (2). T. gondii infection can also be acquired via ingestion of undercooked meat containing the parasite cysts (3). Infection in immunocompromised patients may lead to encephalopathy, coma, and death (4) and in pregnant women, can lead to abortion and congenital defects in the newly born infant (5). T. gondii can also cause abortion and infertility in farm animals (6). Given the significant impact of toxoplasmosis on the health of humans and animals, and the challenges to develop a vaccine for humans (7–9), enhanced understanding of the molecular basis of T. gondii pathogenicity may ultimately lead to the discovery of new treatment options.

RNA modifications and RNA modification enzymes (known as writers) are involved in many biological and pathological processes and play various roles across different domains of life (10–12). RNA modifications are dynamic and key players in the regulation of gene expression in response to an ever-changing cellular microenvironment. Three classes of molecules are involved in RNA modifications: “writers” (catalyze the deposition of specific modifications), “erasers” (catalyze the removal of specific modifications), and “readers” (for recognition and binding of the modified nucleotides) (13). Understanding RNA modifications in parasites, such as *Plasmodium* (14), Leishmania donovani (15), Trypanosoma brucei (16), and *Echinococcus granulosa* (17), has attracted recent attention due to the importance of the regulatory roles of RNA modifications in parasite physiology and development. RNA modifications contribute to stabilizing parasite RNA structures (15) and mediate pathogenicity and host responses to infection (18).

Currently, over 170 different types of posttranscriptional modifications have been identified in different types of RNAs and have energized the growing field of “epitranscriptomics” (19, 20). The mRNA includes different epigenetic modifications which can regulate mRNA stability, splicing, and gene expression (19). The methylation of adenosine at position 6, known as *N*^6^-modification methyladenosine (m^6^A), is the most abundant modification in mRNA and long intergenic ncRNAs (lincRNAs) as well as found in primary miRNA and rRNA (21, 22). m^6^A can regulate ncRNAs via processing and maturation of pri-miRNAs (23), stabilizing circRNAs (24), and regulating the interaction between lncRNAs and proteins (25). The role of m^6^A in T. gondii has recently been reported, where deletion of the m^6^A writer components METTL3 and WTAP causes arrest of parasite replication (26).

T. gondii is a genetically diverse organism, encompassing three main genotypes, namely, type I (e.g., RH strain), type II (e.g., PRU strain and ME49 strain), and type III (e.g., VEG strain and CTG strain), whose virulence varies significantly in mice (27). A previous study showed that avirulent isolates of T. gondii exhibit a higher rate of tRNA cleavage than virulent strains (28). Thus, unravelling the differences in RNA modifications between T. gondii strains which differ significantly with respect to their virulence is of particular importance as it may provide a key to hitherto undiscovered RNA modifications that may underpin the regulatory mechanisms of virulence.

Therefore, in the present study we used a liquid chromatography-tandem mass spectrometry (LC-MS/MS) approach to analyze the spectrum of RNA modifications in T. gondii strains RH, PRU, and VEG, belonging to different genotypes I, II, and III, respectively. We also examined the correlation between RNA modifications and T. gondii genotype-specific virulence traits. We tested the hypothesis that the patterns and diversity of RNA modifications would differ as a function of T. gondii virulence. Our comparative and systematic investigation provided evidence that different types of RNA modifications exist among T. gondii strains of distinct virulence backgrounds. The results offered baseline data for further investigations of the role of RNA modifications in T. gondii infection biology.

## RESULTS

### Genotype-specific RNA modification signatures in T. gondii tRNA and sncRNA.

We determined the patterns of modifications in tRNA (~80 nt fragments) and sncRNA (17 to 50 nt fragments) between T. gondii type I (RH strain), II (PRU strain), and III (VEG strain) using LC-MS/MS-based approach (29). Our analysis identified 22 types of RNA modifications in both tRNA and sncRNA (Table 1). However, due to the low abundance of four RNA modifications (Um, m^5^Um, ac^4^C, and hm^5^C), only 18 types of tRNA and sncRNA modifications were consistently quantified, and thus, shown in the results.

**TABLE 1:** List of 22 posttranscriptional chemical modifications analyzed in T. gondii strains, including four unmodified ribonucleosides (A, U, C, G)

Abbreviation	Name
A	Adenosine
m^1^A	N1-Methyladenosine
I	Inosine
Im	2’-O-Methylinosine
m^6^A	N6-Methyladenosine
Am	2’-O-Methyladenosine
m^1^I	1-Methylinosine
U	Uridine
m^3^U	3-Methyluridine
m^5^U	5-Methyluridine
Um	2’-O-Methyluridine
m^5^Um	5,2’-O-Dimethyluridine
Ψ	Pseudouridine
C	Cytidine
Cm	2’-O-Methylcytidine
ac^4^c	N4-Acetylcytidine
m^5^C	5-Methylcytidine
m^3^C	3-Methylcytidine
hm^5^C	5-Hydroxymethylcytidine
G	Guanosine
m^2^_2_G	N2,N2-Dimethylguanosine
m^2^G	N2-Methylguanosine
Gm	2’-O-Methylguanosine
m^1^G	1-Methylguanosine
m^7^G	7-Methylguanosine
m^2^_2_^7^G	N2,N2,7-Trimethylguanosine

**FIG 1:**
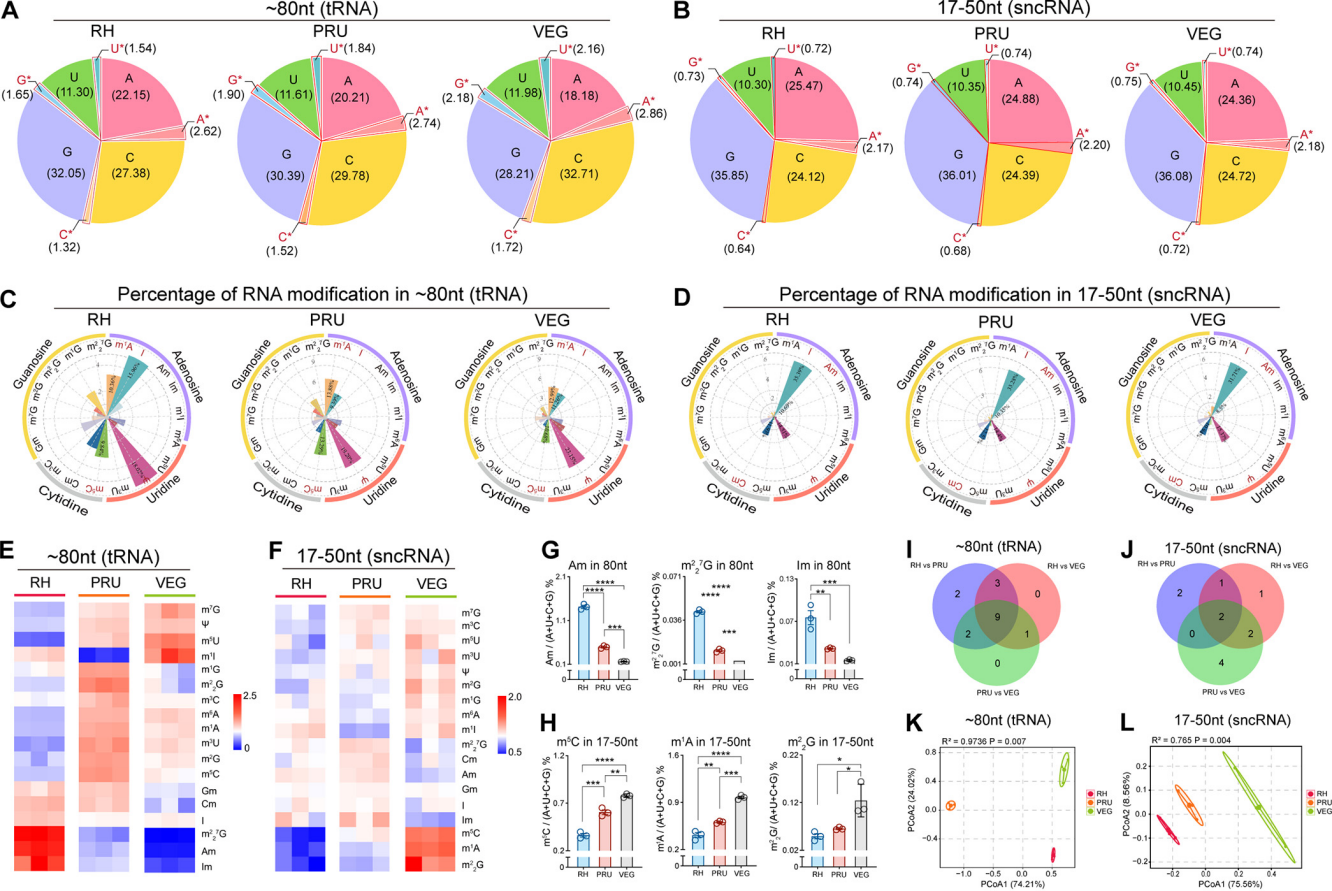
Modifications of tRNA and sncRNA in T. gondii strains RH, PRU, and VEG. Relative percentage of the modified (red) and unmodified (black) four nucleotides (Adenine, Uracil, Cytosine, and Guanine) in tRNA (A) and sncRNA (B). The relative proportion of the detected modifications in tRNA (C) and sncRNA (D). The sum of all RNA modifications was considered 100 and the percentages were calculated from the average of the modifications. Heat maps representing hierarchical cluster analysis showing the expression levels of tRNA modifications (E) and sncRNA modifications (F). Representatives of tRNA modifications (G) and sncRNA modifications (H) with the most differential abundance among the three T. gondii strains. Error bars represent SEM. Student's *t* test was used to determine significance. *, *P* < 0.05; **, *P* < 0.01; ***, *P* < 0.001; ****, *P* < 0.0001. (I to J) Venn diagrams showing the number of the overlapped and unique RNA modifications with differential abundance in each of the three comparison groups: RH strain versus PRU strain, RH strain versus VEG strain, and PRU strain versus VEG strain. (K to L) Principal coordinates analysis (PCoA) plot of tRNA and sncRNA modifications. The abundance of 18 RNA modifications among the three strains was analyzed (*n *= 3 per strain).

The modified ratio of adenosine (A), uridine (U), cytidine (C), and guanosine (G) was calculated according to the levels of RNA modification. The result showed that tRNA exhibited more modifications than sncRNA, where 1–3% of each nucleotide was modified in tRNA, while < 1% of each nucleotide was modified in sncRNA, except A nucleotide (>2%) in the three T. gondii strains (Fig. 1A and B). As shown in Fig. 1C, Ψ was the most marked tRNA modification in the three T. gondii strains, followed by I in RH strain and m^1^A in PRU and VEG strains. In contrast, I was the most marked RNA modification in sncRNA in the three T. gondii strains. Notably, Ψ, I, m^1^A, and m^5^C accounted for over 50% of the modified nucleotides in tRNA, whereas I, Ψ, Cm, and Am represented more than half of the quantified modifications in sncRNA (Fig. 1D). Interestingly, the distribution pattern of the quantified RNA modification percentage was different in tRNA but was similar in sncRNA between T. gondii strains (Fig. 1C and D), indicating that mechanisms for the control of tRNA modification frequency may be strain (genotype) specific.

Further clustering analysis revealed significant differences in the relative expression levels of the quantified tRNA and sncRNA modifications between T. gondii strains (Fig. 1E and F). The tRNA modification pattern in the virulent RH strain had the most distinct pattern compared with the other two less virulent strains (Fig. 1E), particularly the cluster, including tRNA modification m^2^_2_^7^G, Am, and Im whose abundance was proportional to the level of T. gondii virulence. The highest expression level of Am, m^2^_2_^7^G, and Im was detected in RH strain, followed by the less virulent PRU strain and avirulent VEG strain (Fig. 1G). Some sncRNA modifications, such as m^5^C, m^1^A, and m^2^_2_G, were also distinctly expressed between the three strains. However, the expression levels were inversely proportional to the parasite virulence, where the highest expression was detected in VEG strain, followed by PRU strain and the lowest expression was detected in RH strain (Fig. 1H).

Differentially abundant RNA modifications were derived by comparing two T. gondii strains of different virulence backgrounds. According to Venn diagram analysis, nine tRNA modifications (m^1^A, Am, m^1^I, m^5^U, Cm, m^7^G, m^2^G, m^2^_2_G, and m^2^_2_^7^G) and two sncRNA modifications (m^1^A and m^5^C) were expressed at significantly different amounts between RH, PRU, and VEG strains (Fig. 1I and J). Two differentially abundant tRNA modifications (Ψ and m^3^U) were uniquely detected between RH and PRU, whereas two (Ψ and m^3^C), one (Gm), and four (Am, Im, m1G, and m^2^_2_^7^G) differentially abundant sncRNA modifications were detected between RH versus PRU, RH versus VEG, and PRU versus VEG, respectively. PCoA plots showed a strain-specific RNA modification patterns in tRNA and sncRNA, which clearly separated T. gondii strains into three distinct clusters (Fig. 1K and L).

### Linear correlations of different RNA modifications in T. gondii.

**FIG 2:**
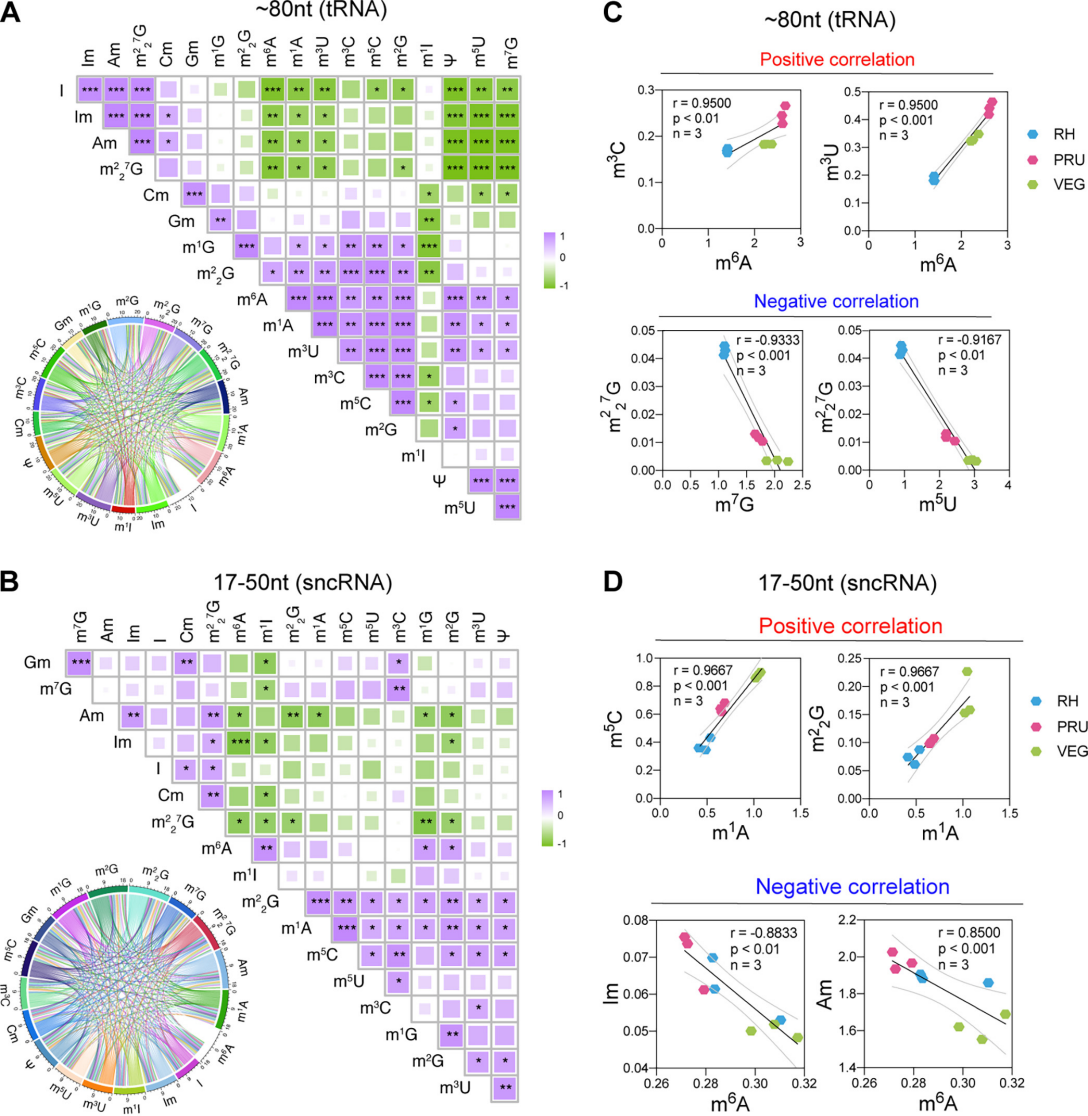
The correlations between different T. gondii RNA modifications. Correlation matrices and chord visualization representing the correlation patterns of tRNA modifications (A) and sncRNA modifications (B). Representatives of the linear correlations detected in tRNA modifications (C) and sncRNA modifications (D). *, *P* < 0.05; **, *P* < 0.01; ***, *P* < 0.001; ****, *P* < 0.0001.

The correlation matrices and chord diagrams showed that many RNA modifications are simultaneously expressed in tRNA (Fig. 2A). and sncRNA (Fig. 2B) in the three T. gondii strains. Among all RNA modifications, linear correlations between some modifications were tRNA-specific, such as m^6^A with m^1^A and m^3^U, and m^2^_2_^7^G with m^7^G and m^5^U (Fig. 2C). We also detected co-expression between specific sncRNA modifications. For example, m^1^A exhibited significant positive linear correlation with m^5^C and m^2^_2_G, whereas m^6^A had a significant negative linear correlation with Am and Im (Fig. 2D). These results show that correlations between modifications of the same RNA class (tRNA or sncRNA) do occur.

### RNA modifications correlate with the expression of T. gondii virulence proteins.

As a strict obligate intracellular parasite, T. gondii possesses an impressive spectrum of effector molecules that enables this parasite to interfere with and hijack the host cell machinery to survive and grow inside the host cell. Some of these effectors are produced by specialized organelles, such as micronemes (MICs) (30), rhoptries (ROPs), and dense granules (GRAs) (31, 32). MICs play roles in the motility and adhesion of T. gondii, while ROPs and GRAs enable the parasite to evade and modulate host immune defences. RNA modifications have been shown to play a role in mediating T. gondii interaction with the host cell. For example, deletion of the m^6^A components WTAP and METTL3 causes complete inhibition of parasite replication (26). Additionally, most of Ψ in tRNA and mRNA of T. gondii relies on pseudouridine synthase (TgPUS1), which plays a role in the differentiation of T. gondii from acute to chronic, suggesting its important role in mediating the host-pathogen interaction (33).

**FIG 3:**
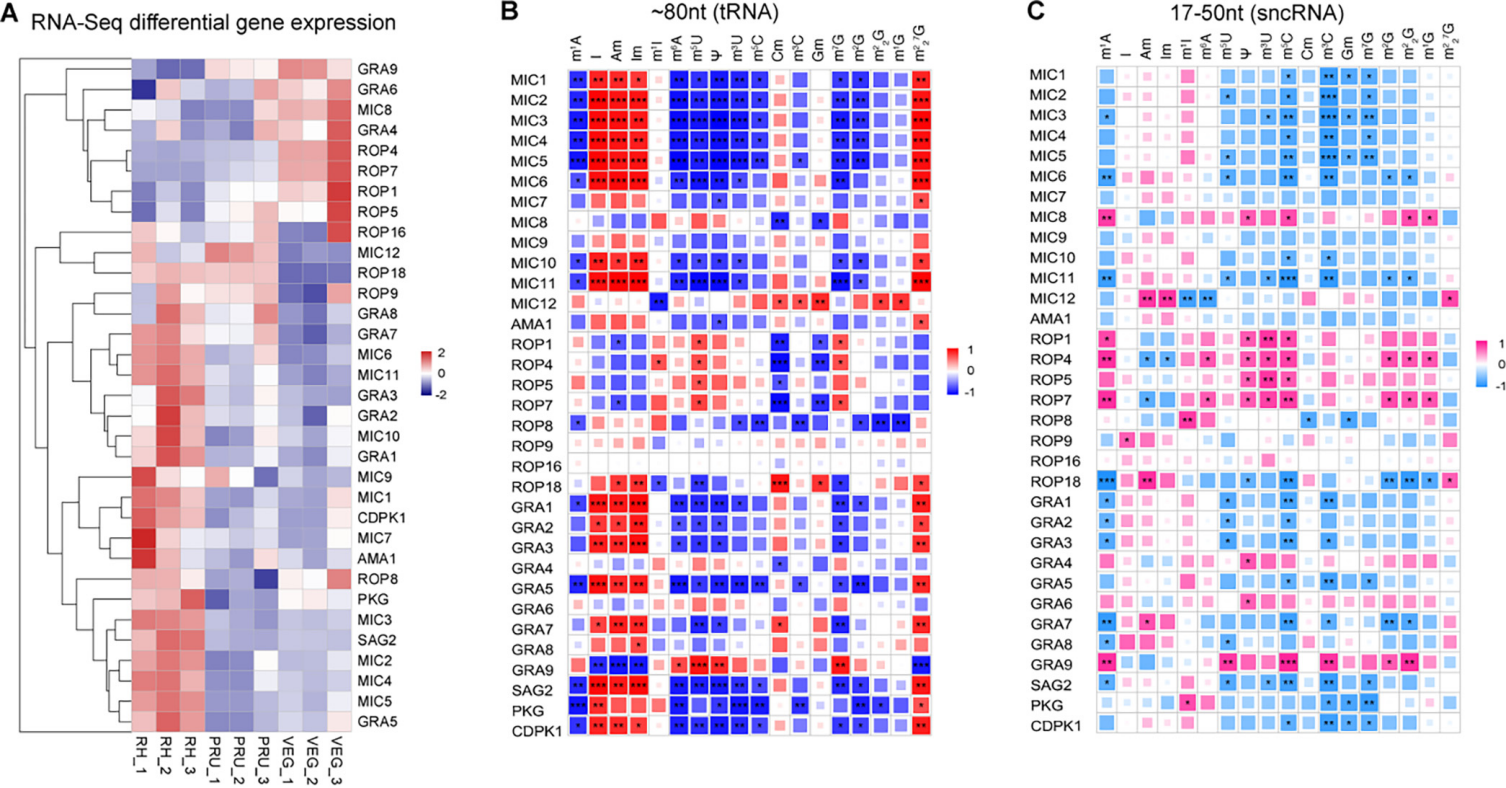
Correlations of the expression levels of virulence proteins among T. gondii strains and of tRNA and sncRNA modification levels with virulence proteins. (A) Heat maps representing color-coded log_2_ ratios of differentially abundant transcripts of T. gondii virulence proteins of T. gondii strains (RH, PRU, and VEG). (B and C) Heat maps showing the correlations between tRNA modifications and virulence proteins (B) and sncRNA modifications and virulence proteins (C). *, *P* < 0.05; **, *P* < 0.01; ***, *P* < 0.001; ****, *P* < 0.0001.

To investigate the potential role of RNA modifications in T. gondii virulence, we investigated the correlation between the parasite virulence proteins and RNA modifications. We used LC-MS/MS approach and next-generation sequencing to quantify the expression of RNA modifications (Table S1) and genes encoding virulence-related proteins (Table 2; Fig. 3A). Quantitation from the entire RNAseq experiment is provided in Table S2. We detected marked associations between many tRNA modifications and the expression levels of genes encoding virulence proteins in all three T. gondii strains (Fig. 3B). For example, the tRNA modification m^6^A was negatively correlated with MIC1-MIC6, MIC10-MIC11, GRA1-3, GRA5, SAG2, PKG, and CDPK1, but was positively correlated with GRA9. Additionally, various RNA modifications exhibited various correlations with different T. gondii proteins. For example, m^1^A, m^6^A, m^5^U, Ψ, m^3^U, m^5^C, m^7^G, and m^2^G were negatively correlated with some virulence proteins (e.g., MIC1-6); most of those proteins had positive correlation with I, Am, Im, and m^2^_2_^7^G. We also analyzed the correlation of sncRNA modifications with T. gondii virulence proteins and found moderate correlations between sncRNA modification patterns and T. gondii virulence factors (Fig. 3C). The only significant negative correlations were detected between Ψ, m^3^U, and m^5^C, and ROP1, ROP4, ROP5, and ROP7 (Fig. 3C).

**TABLE 2:** The gene expression levels (by FPKM values) of virulence-associated proteins in three T. gondii strains obtained by RNA-sequencing

	T. gondii genotype I			T. gondii genotype II			T. gondii genotype III		
Proteins	RH_1	RH_2	RH_3	PRU_1	PRU_2	PRU_3	VEG_1	VEG_2	VEG_3
MIC1	742,20	691,76	586,63	406,09	397,44	459,82	414,23	407,55	552,20
MIC2	1612,68	1829,70	1689,79	864,34	887,59	1210,56	999,26	1008,88	1123,36
MIC3	393,37	363,63	296,67	71.85	60.82	51.75	73.43	69.58	78.76
MIC4	485,71	503,78	387,07	191,61	189,69	266,26	235,93	222,00	239,04
MIC5	1877,62	2289,03	1992,45	743,75	747,82	858,77	938,54	826,90	1068,63
MIC6	458,09	486,41	431,34	293,88	305,28	382,36	266,86	270,98	308,66
MIC7	380,12	314,92	293,98	290,84	284,93	290,44	274,17	274,55	300,14
MIC8	283,79	275,72	245,90	250,43	249,31	304,99	323,68	318,08	360,52
MIC9	139,63	95.59	87.13	108,07	87.43	63.89	79.14	71.70	74.98
MIC10	1595,01	2485,11	1748,25	1034,88	1097,62	1487,47	1262,61	1122,16	1380,74
MIC11	1135,84	1297,94	1034,08	712,62	652,48	770,41	595,76	544,44	614,00
MIC12	50.20	33.84	36.42	59.37	54.89	48.59	27.40	29.71	28.72
AMA1	1100,79	900,04	774,68	740,93	706,45	850,24	767,37	714,62	771,94
ROP1	105,24	137,46	109,72	152,89	166,73	165,73	206,38	194,89	387,66
ROP4	37.16	35.80	38.90	44.15	45.12	53.56	157,05	154,52	310,25
ROP5	53.06	71.43	56.25	75.05	80.65	94.89	78.73	77.15	135,99
ROP7	114,28	113,79	111,86	139,60	156,77	167,39	374,36	365,67	680,37
ROP8	154,55	153,87	115,43	71.84	75.59	48.30	112,77	104,30	191,24
ROP9	125,27	157,48	137,17	141,42	141,90	149,76	112,80	108,62	155,01
ROP16	34.73	31.01	26.72	28.45	29.70	35.63	23.61	23.28	46.55
ROP18	46.72	76.82	59.65	47.45	55.19	54.06	0,02	0,14	0,28
GRA1	5856,57	8982,31	7109,92	3993,54	4038,92	4835,14	3911,45	3553,69	4749,21
GRA2	2863,13	4220,26	3288,61	2692,43	2615,24	2822,64	2568,24	2172,37	2874,98
GRA3	605,98	1191,87	1032,24	537,74	495,44	649,20	500,09	456,57	451,86
GRA4	565,55	655,76	546,27	560,72	532,24	682,80	660,92	617,55	770,56
GRA5	2742,94	4505,21	3322,54	1139,32	1151,59	1406,29	1620,10	1469,45	2189,75
GRA6	588,98	959,83	788,02	739,51	764,11	1032,38	944,93	885,45	1087,05
GRA7	1128,41	1177,73	958,94	870,25	857,30	1031,65	694,87	642,50	751,05
GRA8	419,79	747,90	592,12	460,01	476,01	686,78	378,49	346,22	433,64
GRA9	131,61	106,49	108,17	214,63	200,35	193,16	302,57	290,22	209,33
SAG2	8,70	20.59	18.03	1,60	1,27	1,22	1,87	1,81	1,73
PKG	24.10	24.42	26.32	20.39	21.49	21.30	23.06	23.34	22.64
CDPK1	111,68	98.80	92.93	67.91	73.07	78.57	69.83	69.67	84.58

## DISCUSSION

RNA modifications are involved in multiple cellular and biological processes in prokaryotes and eukaryotes (10, 34). In the present study, LC-MS/MS-based approach was used for comprehensive profiling of RNA modifications in T. gondii strains RH, PRU, and VEG. We analyzed 22 types of RNA modifications, which are commonly identified in tRNA and sncRNA. Of those, Um, m^5^Um, ac^4^C, and hm^5^C were not quantified in tRNAs or sncRNAs of any of the three T. gondii strains, likely attributed to low expression level.

Over 170 types of RNA modifications have been identified across prokaryotes, protista, and fungi (35). In the present study, only 22 types of modified nucleobase standards were used for detection of RNA modifications as previously described (29), limiting the discovery of more RNA modifications in T. gondii strains.

Compared with other RNA classes, tRNA has more modifications, and are involved in regulating tRNA stability, mRNA translation, and protein synthesis (36, 37). The modified nucleotides in tRNA are nearly 10 time more (~17% modified residues) than in the other types of RNAs (1% to 2% modified residues) (38). In agreement with previous studies, the present study revealed that tRNA had more modifications than sncRNA, where 1% to 3% of each nucleotide in tRNA was modified compared with <1% in sncRNA, except A nucleotide (>2%). Interestingly, pseudouridine (Ψ), which exists in all three domains of life (38), was the most abundant tRNA modification in the three T. gondii strains, suggesting its crucial role in T. gondii biology. This result is consistent with a previous study showing that Ψ is one of the most abundant RNA modifications affecting RNA metabolism in T. gondii and that pseudouridine synthase is essential for parasite differentiation (33).

Our analysis revealed a significant diversity of tRNA and sncRNA modification signatures between T. gondii strains with different genotypes. PCA showed that tRNA and sncRNA modification patterns clearly discriminate between the three T. gondii strains. The distribution patterns of the quantified RNA modifications were more diverse in tRNA compared with sncRNA between T. gondii strains, indicating that mechanisms controlling tRNA modification frequency may be dictated by the parasite genotype. Nine modifications were differentially abundant between all three strains in tRNA compared with only two differentially abundant modifications in sncRNA, suggesting that tRNA is more vulnerable for modifications than sncRNA. Interestingly, RH versus PRU had the highest number of tRNA modifications, but the lowest number of sncRNA modifications. Additionally, the expression of tRNA modifications m^2^_2_^7^G, Am, and Im in RH virulent strain (genotype I) was significantly higher than that of the less virulent strain PRU (genotype II) and avirulent strain VEG (genotype III).

Altered levels of tRNA modifications in yeasts can promote an efficient stress response (39). A previous study showed that modification of 2’-O-methylinosine (Im) in yeast mRNA is involved in the yeast response to environmental stress (40). Therefore, the high level of Im in RH strain compared to the less virulent PRU strain and the avirulent VEG strain suggests its potential involvement in stress response of T. gondii. Alterations of tRNA modifications can affect codon decoding fidelity (41), tRNA structure stability (42), and the generation of various types of tRNA-derived small noncoding RNAs (tsRNAs) (43), which play a role in cell transcriptional and translational control in response to stress (44).

Some sncRNA modifications such as m^5^C, m^1^A, and m^2^_2_G were highly expressed in the avirulent VEG strain, compared to the moderately virulent PRU strain and the virulent RH strain. MicroRNA profiling of different T. gondii genotypes revealed both shared and strain-specific microRNAs (45), which may contribute to strain-specific modifications of sncRNA signature. tsRNA fragment, a novel sncRNA type, has been reported in T. gondii and its expression is related to the parasite growth (28). The level of tsRNA is significantly higher in bradyzoite and sporozoite stages of T. gondii compared to the fast-growing tachyzoites. We speculate that differences in the production of tsRNA between the three T. gondii strains may contribute to the differences in the sncRNA modification signature.

Our results revealed linear correlations between various modifications of the same RNA class (tRNA or sncRNA). A previous study in mice detected a correlation between the level of sperm tsRNA m^5^C and the level of sncRNA m^2^G (46). The interaction between tRNA and mRNA modifications has also been reported (47). Whether there are interactions and cross-talks between the different types of RNA modifications identified in the present study remains to be investigated.

T. gondii is defined by three main clonal lineages which differ significantly in virulence capacity mediated by the expression of effector proteins secreted by the specific organelles MICs, ROPs, and GRAs (48–50). Revealing the differential expression of RNA modifications across different T. gondii genotypes is therefore important for broad understanding of the role of RNA modifications in host-parasite interaction. We examined the correlation between expression of genes encoding virulence-related proteins and RNA modification levels among strains representatives of the three main T. gondii genotypes with possible implications of RNA modifications in the expression of virulence factors. The results revealed correlations between specific RNA modification patterns and virulence gene expression patterns. Although our data did not demonstrate a causal link between RNA modifications and T. gondii virulence, proteins included in the correlation analysis (Table 2) such as GRA12 and ROP18 are established virulence factors of T. gondii (48–50).

Given that the expression of virulence factor correlates with parasite virulence, and the virulence gene expression patterns correlate with specific RNA modification patterns, we speculate that RNA modifications may contribute to parasite virulence. Although the expression level of T. gondii virulence proteins is tightly regulated, the molecular mechanisms controlling their regulation remain poorly understood. Various RNA elements operate at different levels of gene expression, ranging from the regulation of transcription and translation, dictating RNA conformation, RNA stability, and modulation of protein binding to RNAs/DNAs as well as activity. Further studies are thus needed to investigate whether RNA modifications can influence any of the aforementioned RNA-mediated processes that regulate the transcription or translation of proteins responsible for the expression of virulence-associated traits in T. gondii.

In conclusion, the present study characterized the modified four nucleotides in T. gondii, identified genotype-specific RNA modification patterns, and detected correlations between RNA modifications of the same RNA class, and between RNA modifications and gene expression of virulence proteins. The discovery of RNA modification patterns distinctive T. gondii strains with different virulence properties should facilitate further investigations to obtain more insight into the molecular mechanisms underlying RNA-based control of virulence gene expression in T. gondii. More research is also needed to investigate whether the three archetypical T. gondii haplotypes encode different copy numbers of specific tRNA genes/species.

## MATERIALS AND METHODS

### Parasite strains.

*Toxoplasma* strains belonging to three T. gondii genotypes, RH strain (type I), PRU strain (type II), and VEG strain (type III), were provided by Dr. Jin-Lei Wang, Lanzhou Veterinary Research Institute. The growth of the tachyzoite stage of these T. gondii strains in monolayers of human foreskin fibroblasts (HFFs) was performed as previously described (48). Heavily infected HFFs were scraped off and passed through 27-gauge needles. The released tachyzoites were purified using a 5 μm Millipore filter and kept in liquid nitrogen until use. The analysis was performed on three biological replicates per T. gondii strain.

### Isolation of ~80 nt and 17 to 50 nt RNA fragments.

The isolation of ~80 nt and 17 to 50 nt RNA fragments was performed according to the manufacturer’s instructions. Briefly, 1 mL RNAiso Plus (TaKaRa, Osaka, Japan) was added to a 1.5 mL microcentrifuge tube containing 1 × 10^6^ T. gondii tachyzoites. The mixture was vortexed vigorously and incubated at room temperature for 5 min. Then, 200 μL of chloroform was added to the sample, followed by vortexing and incubation at room temperature for 10 min. Then, the sample was centrifuged at 12,000 × *g* for 15 min at 4°C. The supernatant was transferred to a new microcentrifuge tube, mixed with an equal volume of isopropanol, incubated at room temperature for 10 min, centrifuged at 12,000 × *g* for 15 min at 4°C, and finally washed with 75% ethanol to dissolve the RNA pellet in RNase-free water. The isolated RNA was electrophoresed in 15% urea-PAGE at constant pressure (200 v) for 1 h in 1× TBE buffer (Invitrogen, Waltham, MA, USA). The gel was stained with SYBR GOLD (Invitrogen, Waltham, MA, USA), and based on the position of the RNA maker (NEB #N2102, #N0364 Ipswich, MA, USA), RNA fragments of ~80 nt and 17 to 50 nt were extracted from the gel as previously described (46).

### Quantitative analysis of RNA modifications by LC-MS/MS.

We used LC-MS/MS approach because mass spectrometry methods have been powerful tools for identification and quantification of RNA modifications (51–53). The isolated RNAs were digested into mononucleotides as previously described (46). Then, 100 ng of RNAs were digested in 30 μL reaction system containing 3 μL 10× RNA hydrolysis buffer (2,500 mM Tirs-HCl, pH 8.0; 50 mM MgCl_2_ and 5 mg/mL BSA), 1 IU benzonase (Sigma-Aldrich, St. Louis, MO, USA), 0.2 IU alkaline phosphatase (Sigma-Aldrich, St. Louis, MO, USA), and 0.05 IU phosphodiesterase I (Thermo Fisher Scientific, Grand Island, NY, USA) at 37°C for 3 h. Then, the enzymes in digestion mixture were removed using a Nanosep 3K spin filter (Pall Corporation, Ann Arbor, MI, USA). Before transferring the digested RNA samples, the spin filter was rinsed twice with 400 μL Watsons distilled water at 14,000 × *g* for 15 min at room temperature. The processed RNAs were stored at −20°C for LC-MS/MS analysis. The LC-MS/MS-based RNA modification detection was performed using waters ACQUITY-UPLC I-class carrying Xevo-TQ-S mass spectrometry system (46). The expression level of each RNA modification was quantified according to a standard curve and RNA modification relative quantification methods previously described (46).

### RNA-sequencing analysis.

We used next-generation sequencing to obtain the transcriptomes of genes associated with virulence properties in T. gondii strains RH, PRU, and VEG. The Nanodrop 2000/2000c spectrophotometer was used to determine the concentration and quality of RNA extracted from all samples (Thermo Fisher Scientific, USA). The RNA libraries were prepared from 1 μg total RNA of each T. gondii sample using the NEBNext Ultra RNA Library Preparation Kit (Illumina, NEB, USA) in accordance with the manufacturer’s recommendations. Each library was sequenced on the Illumina HiSeq 2500 (Illumina, San Diego, CA, USA). Adapter sequences and low-quality reads were removed using Trimomatic (v.0.39) (54). The filtered reads were aligned onto T. gondii reference genome (ftp://ftp.ensemblgenomes.org/pub/release-31/protists//fasta/toxoplasma_gondii/dna/) using ersion 2.0 with default parameters (55). The HTSeq software was used to count the read numbers mapped into each gene and the fragments per kilobase of transcript per million mapped reads (FPKM) was used to measure the expression level of mRNAs in each sample.

### Analysis of RNA modifications and correlations.

Statistical analyses were performed using GraphPad Prism 8 (GraphPad Software Inc., CA, USA). Differences in the levels of ribonucleosides in different strains were examined by Student's *t* test and one-way ANOVA with multiple comparisons. Data are presented as the means ± standard error of means (SEM) of three independent replicates. *P* values < 0.05 were considered statistically significant. Linear correlation analysis was performed to study the correlation between RNA modifications or between RNA modifications and RNA modification enzymes. The transcriptomic data of selected genes encoding virulence-related proteins were used to explore the correlation between RNA modifications and virulence-related proteins. Hierarchical clustering heatmaps and correlation matrices were obtained using the R package corrplot and correlations were calculated based on the mean values of the measured modifications using the “Spearman” method. Correlation analysis was used to assess the degree of covariance among the various RNA modifications for each parasite strain, with correlation coefficients calculated using GraphPad Prism 8. Principal coordinates analysis (PCoA) of RNA modification levels was performed using the R package scatterplot3d. The PCoA and chord plots were generated using the R packages scatterplot3d and circlize, respectively.

### Data availability.

RNA-seq reads for the T. gondii have been submitted to NCBI under the accession number PRJNA932999.
