# Supplementary material for: Unravelling Strain-Specific Modifications of Toxoplasma gondii tRNA and sncRNA Using LC-MS/MS
Source: Microbiol Spectr. 2023 Apr 10;11(3):e03564-22. doi: 10.1128/spectrum.03564-22 (PMC10269570; doi:10.1128/spectrum.03564-22)
Supplement: Supplemental file 1 — Table S1. Download spectrum.03564-22-s0001.pdf, PDF file, 0.1 MB [file spectrum.03564-22-s0001.pdf]

Table S1. Quantitative values of 18 RNA modifications in different regions of RNA of three *T. gondii* genotypes.

**Sample ID**	A	m^1A	I	Am	Im	m^1I	m^6A	U	m^5U	Ψ	m^3U	C	m^5C	Cm	m^3C	G	Gm	m^7G	m^2G	m^2_2G	m^1G	$m^{2,7}_2G$
RH_80nt_1	94.902	3.163	4.752	1.352	0.059	0.264	1.420	48.648	0.942	5.394	0.180	117.335	2.988	2.382	0.174	133.075	1.967	1.119	0.775	1.064	1.983	0.043
RH_80nt_2	96.017	3.164	4.995	1.477	0.093	0.281	1.413	48.627	0.907	5.502	0.197	114.321	3.005	2.476	0.164	141.036	1.793	1.107	0.634	1.064	2.165	0.045
RH_80nt_3	93.787	3.165	4.874	1.407	0.074	0.298	1.407	47.951	0.873	5.610	0.181	120.349	3.021	2.570	0.169	137.912	2.107	1.094	0.811	1.064	2.347	0.041
PRU_80nt_1	74.350	5.941	4.011	0.495	0.031	0.069	2.609	55.248	2.195	8.168	0.442	154.618	5.816	2.478	0.227	118.389	2.137	1.653	1.426	2.060	3.299	0.013
PRU_80nt_2	71.925	6.078	4.109	0.462	0.031	0.092	2.596	54.991	2.190	8.404	0.419	156.944	5.899	2.308	0.245	116.141	2.096	1.712	1.431	2.178	3.264	0.012
PRU_80nt_3	73.137	6.214	4.207	0.528	0.033	0.083	2.667	55.505	2.440	8.640	0.464	153.831	5.733	2.522	0.266	121.932	2.238	1.784	1.421	2.119	3.333	0.010
VEG_80nt_1	62.019	4.542	4.020	0.165	0.016	0.358	2.255	58.975	2.830	8.369	0.327	162.989	4.158	1.446	0.183	116.017	1.514	1.858	0.955	1.368	2.197	0.003
VEG_80nt_2	62.072	4.790	4.234	0.171	0.014	0.525	2.372	58.034	3.051	8.601	0.348	159.774	3.914	1.762	0.183	120.120	1.546	2.241	1.063	1.122	1.883	0.003
VEG_80nt_3	62.046	4.955	4.127	0.159	0.015	0.441	2.208	58.544	2.941	8.485	0.322	161.382	3.671	1.099	0.182	118.068	1.429	2.049	1.034	0.877	1.568	0.004
RH_17_50nt_1	105.378	0.536	5.868	1.905	0.070	0.032	0.283	42.135	0.351	2.486	0.100	99.693	0.433	1.971	0.173	146.188	1.458	0.668	0.210	0.088	0.493	0.090
RH_17_50nt_2	105.296	0.412	6.649	1.883	0.061	0.034	0.284	41.616	0.298	2.593	0.095	98.187	0.360	2.182	0.177	148.742	1.507	0.714	0.211	0.074	0.448	0.098
RH_17_50nt_3	105.214	0.486	6.186	1.860	0.053	0.035	0.310	43.991	0.252	2.591	0.103	101.200	0.346	2.154	0.168	149.596	1.470	0.622	0.261	0.061	0.510	0.091
PRU_17_50_1	100.880	0.656	6.337	1.935	0.074	0.026	0.273	43.983	0.341	2.866	0.117	105.766	0.617	2.362	0.199	152.251	1.539	0.774	0.241	0.100	0.458	0.105
PRU_17_50_2	100.416	0.645	6.158	1.966	0.061	0.027	0.279	43.521	0.390	2.507	0.114	104.028	0.640	2.343	0.210	152.035	1.547	0.788	0.241	0.098	0.457	0.103
PRU_17_50_3	101.345	0.690	6.569	2.026	0.075	0.026	0.271	43.274	0.329	2.873	0.119	104.897	0.682	2.494	0.204	153.991	1.561	0.823	0.239	0.108	0.493	0.102
VEG_17_50_1	97.721	1.047	5.482	1.553	0.052	0.034	0.308	47.866	0.430	3.039	0.128	109.659	0.880	1.918	0.211	145.615	1.507	0.761	0.316	0.227	0.573	0.074
VEG_17_50_2	97.435	1.021	6.340	1.621	0.050	0.031	0.298	46.323	0.382	2.737	0.105	109.612	0.859	2.267	0.203	145.401	1.526	0.767	0.264	0.153	0.495	0.092
VEG_17_50_3	97.149	1.073	6.331	1.690	0.048	0.037	0.317	47.316	0.345	3.022	0.142	109.706	0.901	2.075	0.204	145.830	1.489	0.730	0.293	0.159	0.550	0.087
